# All‐Suture Anchor Fixation for Coronoid Process Fractures via a Transolecranon Fracture Approach

**DOI:** 10.1155/cro/6402842

**Published:** 2026-07-20

**Authors:** Yuxuan Wu, Yang Lu, Jianlan Wang, Jiang Mu, Shuang Zhang

**Affiliations:** ^1^ Department of Orthopedics, Central Hospital of Dalian University of Technology, Dalian, Liaoning, China; ^2^ Department of Medical Intensive Care Unit, Central Hospital of Dalian University of Technology, Dalian, Liaoning, China; ^3^ Department of Traditional Chinese Medicine, Central Hospital of Dalian University of Technology, Dalian, Liaoning, China

**Keywords:** all-suture anchor, bidirectional compressive forces, coronoid process fracture, dual-directional compression, elbow fractures, fracture stabilization, surgical treatment

## Abstract

**Background:**

Coronoid process fractures are a common component of elbow injuries and present significant challenges in surgical management. The all‐suture anchor fixation technique, as an emerging method, offers a novel alternative for stabilizing these fractures. By enabling dual‐directional fixation, this approach provides bidirectional mechanical support, resulting in enhanced stabilization of the fracture fragment.

**Case Presentation:**

A 35‐year‐old male presented with a complex proximal ulnar fracture involving both the coronoid process (O′Driscoll Type III) and the olecranon. Surgical fixation was performed using an all‐suture anchor inserted at the fracture site, with sutures strategically tied to a fixation plate, establishing a stable tension‐band configuration. This construct provided controlled traction and stable three‐dimensional support of the fragment. Postoperative recovery was uneventful, with full restoration of elbow function and no evidence of fragment displacement or complications.

**Conclusion:**

This case illustrates the technical feasibility of the all‐suture anchor technique for coronoid fractures. In cases of complex proximal ulnar fractures, this technique allows for simultaneous fixation of the coronoid and olecranon through a single posterior approach, reducing soft tissue disruption.

## 1. Introduction

Ulnar coronoid process fractures are clinically significant due to their critical role in maintaining elbow joint stability. These fractures often occur in conjunction with elbow dislocation, ligament injuries, and other fractures, particularly in the context of the “terrible triad” injury. If not promptly and properly treated, these injuries can lead to elbow instability, which may result in recurrent dislocations and further damage to the joint [1].

Common treatment options include conservative and surgical approaches. For small, isolated coronoid fractures, conservative management often yields satisfactory results. However, for larger fractures or complex cases associated with other fractures and ligamentous injuries, open reduction and internal fixation (ORIF) is typically the preferred treatment method to restore joint stability and function [2].

The all‐suture anchor is a novel fixation device that has gained widespread use in orthopedic surgeries in recent years. Its principle lies in the formation of a fixation point within the bone using sutures, establishing a stable connection between the soft tissue and bone. Compared with traditional metal screws, all‐suture anchors offer several advantages, including reduced bone removal, minimized risk of displacement, and fewer postoperative complications [3].

In the treatment of tibial spine avulsion fractures, all‐suture anchors have been utilized for arthroscopically assisted reduction and internal fixation. This technique, employing multiple loops of sutures and cortical suspension suture‐locking devices, effectively overcomes the limitations of traditional screw fixation [4]. Similarly, in shoulder labral repairs, the use of all‐suture anchors has demonstrated significant clinical outcomes. Studies indicate that patients undergoing arthroscopic labral repair with all‐suture anchors exhibit improved postoperative shoulder function scores, with athletes achieving favorable recovery of their athletic performance [5]. This technique not only reduces bone removal but also lowers the risk of postoperative synovitis and cartilage damage.

This report presents a case of a coronoid process fracture associated with an olecranon fracture treated with all‐suture anchor fixation via a single posterior approach.

## 2. Case Presentation

A 35‐year‐old male, a practicing dentist, presented with a right elbow injury sustained from a fall during athletic activity. He experienced immediate severe pain, restricted motion, and noticeable deformity and swelling in the affected elbow. Physical examination revealed a closed injury with significant swelling, deformity, and disruption of the bony landmarks of the elbow. However, there were no signs of joint dislocation, and the peripheral neurovascular function was intact. A preliminary diagnosis of an elbow fracture was made, and a temporary cast was applied for stabilization.

**Figure 1:**
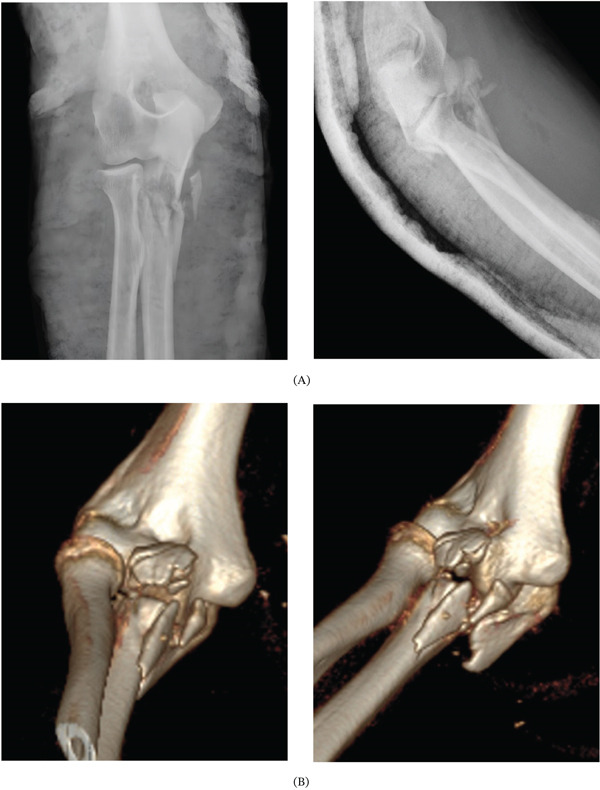
Preoperative radiographic and computed tomography (CT) findings of the right elbow. (A) Anteroposterior and lateral digital radiographs (DR) demonstrating a complex fracture of the proximal ulna involving both the coronoid process and the olecranon, with displacement of the coronoid fragment. (B) Three‐dimensional CT reconstructions providing detailed visualization of the comminuted coronoid process fracture (O′Driscoll Type III) and the associated transolecranon fracture.

Radiographic assessment, including standard anteroposterior and lateral x‐rays, as well as three‐dimensional CT reconstruction, revealed a complex fracture of the proximal ulna (Figure 1). Specifically, there was a large, comminuted fracture of the coronoid process measuring approximately 3 × 3 cm, consistent with an O′Driscoll Type III coronoid process fracture. Additionally, a transverse fracture of the olecranon on the same side was identified.

**Figure 2:**
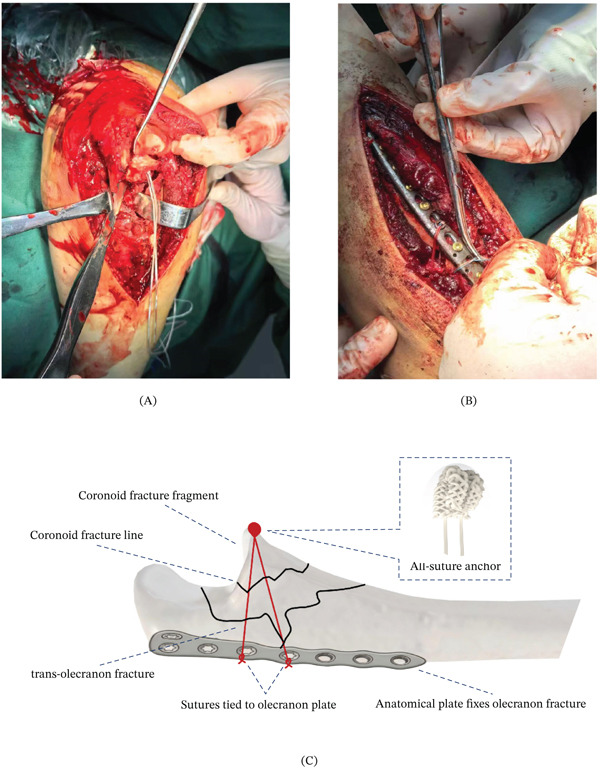
Intraoperative photographs and schematic illustration of the all‐suture anchor fixation technique via a transolecranon approach. (A) Intraoperative image showing placement of the all‐suture anchor into the prepared bone bed of the coronoid fracture fragment, with the tail sutures passed across the fracture line. (B) Intraoperative image showing the tail sutures tied to the dorsal olecranon fixation plate, and the anatomical plate spanning the transolecranon fracture to achieve simultaneous fixation of both the coronoid and olecranon fractures through a single posterior approach. (C) Schematic diagram of the transolecranon approach combined with all‐suture anchor fixation for the coronoid fracture. The coronoid fracture fragment (red marker) is secured by an all‐suture anchor inserted into the fracture bed (inset: magnified view of the anchor). After crossing the fracture line, the anchor′s tail sutures are tied to the olecranon fixation plate, forming a suture tension‐band construct; the plate serves as a posterior fulcrum, and suture tension draws the coronoid fragment against the fracture bed, providing anteroposterior compressive fixation. The anatomical plate spans the transolecranon fracture site and, through the same posterior approach, simultaneously fixes the olecranon fracture.

The patient underwent surgical treatment on the third day postinjury. The procedure was performed under brachial plexus anesthesia, through a single posterior midline approach. The ulnar nerve was carefully identified and protected. By mobilizing the proximal olecranon fragment, a direct “transfracture” view of the coronoid process was obtained without extensive soft tissue stripping. The coronoid fracture fragment was found to be large with relatively low comminution. A single 4.5‐mm Star all‐suture anchor (suture material: ultrahigh molecular weight polyethylene [UHMWPE]) was selected. The anchor was placed centrally in the coronoid fracture bed, with the drill hole oriented perpendicular to the fracture line and directed through the bone immediately anterior to the coronoid process. The two sutures were passed around the fragment and tied to the dorsal olecranon plate. This configuration ensured that the sutures were oriented perpendicular to the fracture line, creating direct compression of the coronoid fragment against the ulnar shaft while neutralizing anterior shear forces. The olecranon was fixed with an anatomical plate. The two high‐strength sutures from the anchor′s tail were passed around and securely tied to the plate holding the olecranon, achieving anterior–posterior fixation (Figure 2).

**Figure 3:**
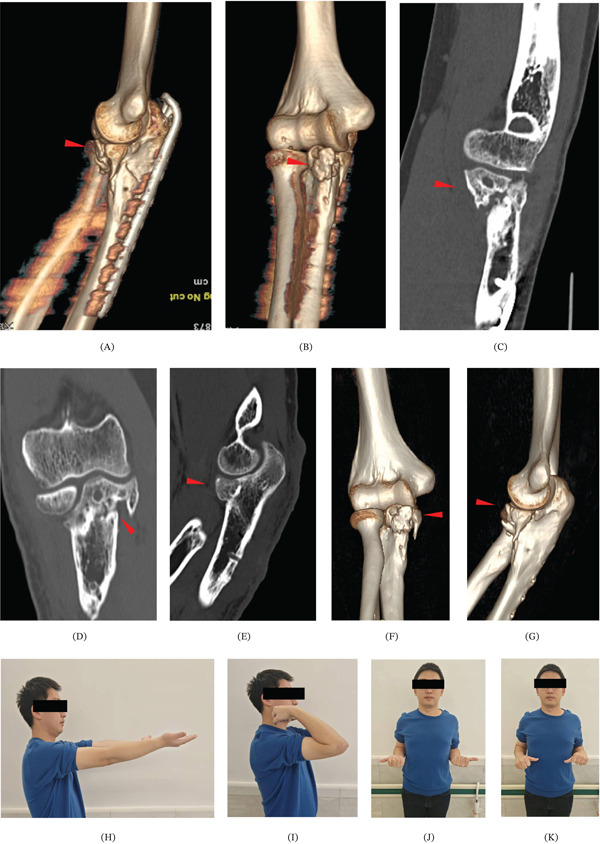
Postoperative follow‐up radiographic and clinical outcomes. (A–C) Three‐dimensional CT reconstructions and sagittal CT image obtained at 3‐month follow‐up, showing the coronoid process fracture site (red arrowheads) with early bone callus formation and the bone tunnel created by the all‐suture anchor. (D–G) Three‐dimensional CT reconstructions and coronal/sagittal CT images obtained at 1‐year follow‐up, after removal of the internal fixation plate, demonstrating complete osseous union of the coronoid process and olecranon fractures (red arrowheads) with restoration of normal humeroulnar joint congruity and no evidence of posttraumatic arthritis, nonunion, or implant‐related complications. (H–K) Clinical photographs at 1‐year follow‐up demonstrating full functional recovery of the elbow, including (H) full extension, (I) full flexion, and (J, K) symmetric forearm pronation and supination compared with the contralateral side.

On the first postoperative day, passive range‐of‐motion exercises were initiated under the guidance of a rehabilitation therapist. Gradual transition to active functional exercises began 1 month postoperatively. The patient was followed up regularly until 3 months postsurgery, at which time the patient had achieved excellent elbow function with normal muscle strength, no pain, or instability, with a Mayo Elbow Performance Score (MEPS) of 95/100 (excellent). Follow‐up CT imaging showed excellent alignment of the coronoid process fracture with clear bone callus formation, indicating satisfactory healing progress; bone healing was assessed using a modified Radiographic Union Score for Fractures (RUFS) based on CT, with a score of 16/16 (complete cortical bridging in all four cortices) at the 1‐year follow‐up (Figure 3). Throughout the perioperative period and follow‐up, no complications such as wound infection, implant failure, ulnar nerve injury, heterotopic ossification, or joint stiffness were observed. At the patient′s request, the internal fixation plate was removed at 1 year postoperatively. CT imaging obtained at that time confirmed complete osseous union of both the coronoid process and olecranon fractures, with restoration of normal humeroulnar joint congruity and no evidence of nonunion, posttraumatic arthritis, or implant‐related complications. Clinical examination at the 1‐year follow‐up demonstrated full, symmetric elbow extension, flexion, pronation, and supination compared with the contralateral side (Figure 3; Table 1).

**Table 1:** Timeline.

Time point	Event
Day 0	Fall during athletic activity; closed proximal ulnar fracture (coronoid + olecranon); temporary cast applied
Day 3	Surgery: transolecranon approach, all‐suture anchor fixation of coronoid fragment, anatomical plate fixation of olecranon
POD 1	Passive range‐of‐motion exercises initiated under rehabilitation therapist guidance
1 month	Transition to active functional exercises
3 months	Excellent elbow function, MEPS 95/100; CT showed satisfactory callus formation and fracture alignment; no complications
1 year	Implant removal; CT confirmed complete osseous union of coronoid and olecranon fractures; full, symmetric functional recovery

## 3. Discussion

Coronoid process fractures are common elbow injuries, with various fixation techniques employed for surgical management, including plate fixation, screw fixation, and traditional suture fixation. Plate fixation is a widely used choice, particularly for complex fractures. The advantage of plate fixation lies in its ability to provide stable fixation and allow for early functional rehabilitation. However, it has some drawbacks, such as the need for multiple incisions, larger surgical trauma, and potential soft tissue damage, which can increase the risk of postoperative complications [6]. Screw fixation is a relatively newer method typically used for simpler fractures. The advantage of screw fixation lies in its minimal invasiveness, faster recovery, and the biocompatibility of titanium. However, screw fixation may not offer the same level of stability as plate fixation, especially when addressing complex fractures [7]. Traditional suture fixation is primarily employed for simple fractures or as an adjunctive fixation technique. Its advantages include simplicity, minimal trauma, and low risk of complications, but it is limited by its strength, making it unsuitable for fractures requiring high stability [8].

The use of all‐suture anchors for the fixation of small fracture fragments offers several advantages, including minimal invasiveness, superior biocompatibility, and optimal matching of fixation strength with bone elasticity. Compared with traditional open surgeries, all‐suture anchor fixation reduces surgical trauma and lowers the risk of postoperative complications. This method is particularly effective in treating complex fractures, such as Pipkin Type I femoral head fractures. The use of absorbable suture anchors provides initial stability, with the double pulley technique further enhancing fracture fixation, achieving an effective stabilization [9]. The fixation strength of all‐suture anchors, in harmony with the elasticity of the bone, plays a crucial role in fracture healing. In studies comparing single‐point and double‐point suture bridge techniques, although there were no significant differences in failure strength or load transfer, the double‐point suture bridge technique showed greater advantage in the initial stability of fracture fragments. This technique effectively reduces fragment displacement under concentrated loading, offering more stable fixation [10].

In the present case, the all‐suture anchor fixation for the coronoid process fracture employed an anterior–posterior suture binding with the fixation plate, applying traction while establishing secure multiplanar fixation. Specifically, by securely tying the anchor′s tail suture to the fixation plate, stable support was provided in multiple directions for the fracture fragment. This construct not only effectively prevented displacement but also ensured the fragment remained in the optimal anatomical position throughout the healing process. Furthermore, when the full‐suture anchor is tightened, the compressive effect induces a force that brings the bone fragments on either side of the fracture closer together, thus achieving a more stable fixation. This fixation mechanism is analogous to the tension band principle, where upon effective tightening, a bidirectional compressive force is applied between the fracture fragments, further stabilizing the fracture site and ensuring more reliable fracture healing. This approach demonstrated notable advantages, especially in patients with large fracture fragments and good bone density. The anterior–posterior suture binding created a stable three‐dimensional fixation structure that provided strong initial stabilization and maintained fragment stability during healing. By applying multidirectional fixation forces, the technique minimized the risks associated with single‐point fixation seen in traditional screw methods, preventing fragment displacement or secondary damage.

From a biomechanical standpoint, all‐suture anchors differ fundamentally from rigid metal or polymer anchors in that the entire implant—both the body and the eyelet through which the sutures pass—is constructed of braided suture material. Once deployed, the anchor body expands and collapses against the surrounding cancellous bone, distributing the pull‐out load over a larger area of the bone‐implant interface rather than concentrating it at a single rigid point. This deformable, friction‐based fixation mode reduces the peak stress transmitted to the surrounding bone and is thought to lower the risk of anchor pull‐out or cut‐through, particularly in fracture fragments with low bone density or limited cortical thickness, such as the coronoid process. In the present construct, tensioning the anchor′s tail sutures against the olecranon plate converts this friction‐based anchor fixation into a suture tension‐band system: The plate acts as a rigid posterior fulcrum, and suture tension is transmitted as a compressive force directed from the bone tunnel toward the fracture bed. This arrangement allows the relatively low stiffness of the suture‐anchor interface to be converted into a clinically effective compressive force across the fracture line, which is the principal biomechanical rationale for selecting an all‐suture anchor, rather than a screw or rigid suture anchor, for this anatomically constrained fragment.

Preoperative CT assessment of the fracture fragment′s size, shape, and the bone quality anterior to the coronoid process ensured adequate bone strength to support the anchor‐based fixation. Intraoperatively, the anchor was precisely placed perpendicular to the fracture line of the fracture, completely traversing the bone anterior to the coronoid process, ensuring that the tail suture effectively applied traction to the fracture fragment, providing uniform stabilization from multiple stress directions. Although the postoperative CT scan at 3 months showed a minor residual gap at the cortical rim, clinical stability was excellent. This radiographic appearance may be attributed to the early stage of healing or minor resorption at the fracture edge, but it did not affect the mechanical integrity of the construct or the patient′s functional recovery, a hypothesis subsequently confirmed at 1‐year follow‐up, when CT performed after implant removal demonstrated complete osseous union with no residual gap.

This case illustrates the feasibility of all‐suture anchor fixation for coronoid process fractures, with excellent postoperative functional recovery. Compared with traditional suture fixation through bone tunnels, the all‐suture anchor minimizes bone loss and simplifies the surgical steps in a deep operative field. Drilling multiple tunnels in a comminuted coronoid process risks further fragmentation, whereas the anchor provides a secure hold with a smaller footprint. Taken together with the 1‐year follow‐up data, these findings suggest that all‐suture anchor fixation is a technically feasible option for coronoid process fractures associated with olecranon fractures; however, given the single‐case design, this conclusion should be regarded as hypothesis‐generating rather than definitive evidence of safety or efficacy.

## 4. Limitations

This report has several limitations. First, as a single‐case design, it cannot establish the efficacy, safety, or generalizability of all‐suture anchor fixation for coronoid process fractures; the findings represent a proof‐of‐concept demonstration of technical feasibility rather than comparative evidence against established fixation methods. Second, although follow‐up was extended to 1 year with implant removal and confirmation of complete osseous union, this duration remains insufficient to exclude late complications such as posttraumatic osteoarthritis, heterotopic ossification, or recurrent instability, which may manifest only after several years. Third, outcome assessment relied on a single clinical evaluator and a single functional scoring instrument (MEPS) without an independent radiologic adjudicator, which may introduce assessment bias. Finally, the favorable result in this patient, who had a large, minimally comminuted coronoid fragment and good bone quality, may not be reproducible in patients with smaller or more comminuted fragments or osteoporotic bone, in whom anchor pull‐out risk may be higher. Larger comparative case series with longer follow‐up are needed to validate the generalizability of these findings.

## 5. Conclusion

This case illustrates the technical feasibility of all‐suture anchor fixation for treating coronoid process fractures, particularly when associated with olecranon fractures. Utilizing the olecranon fracture line as a surgical window allows for a single posterior approach to address both injuries, and the construct maintained stable fixation and satisfactory functional recovery through 1 year of follow‐up, including after implant removal. The key clinical lesson from this case is that, in a comminuted but largely intact coronoid fragment without sufficient bone stock for screw purchase, an all‐suture anchor tensioned against a posterior plate can provide a workable alternative to screw or rigid‐anchor fixation while avoiding an additional anterior approach. This feasibility observation should be confirmed in larger comparative series with longer follow‐up before the technique can be recommended for broader clinical use.

## Author Contributions

Yuxuan Wu, Jiang Mu, and Shuang Zhang performed the surgery, collected clinical data, and contributed to the study design. Yang Lu contributed to data curation, visualization, interpretation of clinical and imaging findings, and critical revision of the manuscript. Jianlan Wang drafted the manuscript, performed language polishing, and revised the content.

## Funding

No funding was received for this manuscript.

## Disclosure

All authors read and approved the final manuscript.

## Ethics Statement

This case report was reviewed by the Ethics Committee of Central Hospital of Dalian University of Technology, which granted an exemption from formal ethical approval for single‐case retrospective case reports (Approval/Exemption No. ZM2025‐024‐01), in accordance with institutional policy and the Declaration of Helsinki. Written informed consent for publication was obtained from the patient, as detailed in the Consent section.

## Consent

Written informed consent was obtained from the patient for participation in this study and for publication of this case report and any accompanying images.

## Conflicts of Interest

The authors declare no conflicts of interest.

## Data Availability

All data generated or analyzed during this study are included in this article.
